# An investigation into inter- and intragenomic variations of graphic genomic signatures

**DOI:** 10.1186/s12859-015-0655-4

**Published:** 2015-08-07

**Authors:** Rallis Karamichalis, Lila Kari, Stavros Konstantinidis, Steffen Kopecki

**Affiliations:** 1 Department of Computer Science, University of Western Ontario, London, ON Canada; 2 Department of Mathematics and Computing Science, Saint Mary’s University, Halifax, NS Canada

**Keywords:** Comparative genomics, Genomic signature, Species classification

## Abstract

**Background:**

Motivated by the general need to identify and classify species based on molecular evidence, genome comparisons have been proposed that are based on measuring mostly Euclidean distances between Chaos Game Representation (CGR) patterns of genomic DNA sequences.

**Results:**

We provide, on an extensive dataset and using several different distances, confirmation of the hypothesis that CGR patterns are preserved along a genomic DNA sequence, and are different for DNA sequences originating from genomes of different species. This finding lends support to the theory that CGRs of genomic sequences can act as *graphic genomic signatures*. In particular, we compare the CGR patterns of over five hundred different 150,000 bp genomic sequences spanning one complete chromosome from each of six organisms, representing all kingdoms of life: *H. sapiens* (Animalia; chromosome 21), *S. cerevisiae* (Fungi; chromosome 4), *A. thaliana* (Plantae; chromosome 1), *P. falciparum* (Protista; chromosome 14), *E. coli* (Bacteria - full genome), and *P. furiosus* (Archaea - full genome). To maximize the diversity within each species, we also analyze the interrelationships within a set of over five hundred 150,000 bp genomic sequences sampled from the entire aforementioned genomes. Lastly, we provide some preliminary evidence of this method’s ability to classify genomic DNA sequences at lower taxonomic levels by comparing sequences sampled from the entire genome of *H. sapiens* (class Mammalia, order Primates) and of *M. musculus* (class Mammalia, order Rodentia), for a total length of approximately 174 million basepairs analyzed. We compute pairwise distances between CGRs of these genomic sequences using six different distances, and construct Molecular Distance Maps, which visualize all sequences as points in a two-dimensional or three-dimensional space, to simultaneously display their interrelationships.

**Conclusion:**

Our analysis confirms, for this dataset, that CGR patterns of DNA sequences from the same genome are in general quantitatively similar, while being different for DNA sequences from genomes of different species. Our assessment of the performance of the six distances analyzed uses three different quality measures and suggests that several distances outperform the Euclidean distance, which has so far been almost exclusively used for such studies.

## Background

Alongside DNA barcoding, [1] and Klee diagrams [2], Chaos Game Representation (CGR) patterns of genomic segments have been proposed as another method for the classification and identification of genomic sequences [3, 4]. The concept of *genomic signature* was first introduced in [5], as being any specific quantitative characteristic of a DNA genomic sequence that is pervasive along the genome of the same organism, while being dissimilar for DNA sequences originating from different organisms. Initial studies [3, 6] suggesting that short fragments of genomic sequences retain most of the characteristics of the genome of origin indicated that such genomic signatures exist. In particular, the Chaos Game Representation (CGR) of a DNA sequence, a graphic representation of its sequence composition, was proposed in [3] as having both the pervasiveness and differentiability properties necessary for it to qualify as a genomic signature. Indeed, CGRs of genomic DNA sequences have been shown to be genome- and species-specific, see, e.g., [3, 4, 6–12]. Note that CGR patterns of mtDNA sequences can be different from those of DNA sequences from the major genome of the same organism, and that large scale quantitative analyses, at all taxonomic levels, of the hypothesis that CGR can play the role of a genomic signature for genomic sequences have not, to our knowledge, been performed. The long term objective of this research is to find out whether CGR can play the role of genomic signature for genomic DNA sequences, and can be used to identify and classify genomic sequences at all taxonomic levels. To this end, the objective of this study is to quantitatively assess the usability of CGR for classification of genomic sequences at the kingdom level, as well as to assess various distances that can be used to compare CGRs of genomic sequences for this purpose.

We first analyze 508 fragments, 150 kbp (kilo base pairs) long, spanning single complete chromosomes of six organisms, each representing a different kingdom: chromosome 21 of *Homo sapiens*, chromosome 4 of *Saccharomyces cerevisiae*, chromosome 1 of *Arabidopsis thaliana*, chromosome 14 of *Plasmodium falciparum*, the genome of *Escherichia coli*, and the genome of *Pyrococcus furiosus*, for a total length of 76,200 kbp analyzed. We analyze the intergenomic and intragenomic variation of CGR genomic signatures of these sequences by using six different distances: Structural Dissimilarity Index (DSSIM) [13], Euclidean distance, Pearson correlation distance [14], Manhattan distance [15], approximated information distance [16], and a distance defined here, based on an idea from computer vision, called *descriptor distance*. For each of the six distances, we visualize the results by computing Molecular Distance Maps, [12], which represent sequences as points in a two-dimensional or three-dimensional space, and thus display all their interrelationships simultaneously. The resulting Molecular Distance Maps show a good clustering, with genomic sequences originating from the same genome being largely grouped together, and separated from sequences belonging to genomes of different organisms. We observe that, in some of the cases where the clustering is suboptimal, the computation of three-dimensional Molecular Distance Maps resolves what appeared to be cluster overlaps in the two-dimensional Molecular Distance Maps. Using the “ground-truth” that sequences from the same genomes should have similar structural characteristics and thus be grouped together, while those from genomes of different organisms should be separated, we assess the six distances by combining three different quality measures: correlation to an idealized cluster distance, silhouette accuracy, and histogram overlap. We conclude that, for this dataset, DSSIM and the descriptor distance perform best according to these measures.

To maximize the diversity within each species, we also analyze a set of 526 fragments, 150 kbp long, sampled from the entire genomes of the aforementioned six organisms, for a total length of 78,900 kbp analyzed. The resulting Molecular Distance Maps are very similar to the ones in the first experiment, and the distance ranking is also the same, confirming the preceding results.

Lastly, we provide some preliminary evidence of this method’s applicability to classifying genomic DNA sequences at lower taxonomic levels by comparing 240 genomic sequences, 150 kbp long, sampled from the entire genome of *Homo sapiens* (class Mammalia, order Primates) with 210 genomic sequences, 150 kbp long, sampled from the entire genome of *Mus musculus* (mouse, class Mammalia, order Rodentia) for an additional length of 67,500 kbp analyzed. While a clear separation of sequences by genome is indeed achieved, we observe that the distance ranking is quite different compared to the previous two experiments, indicating that different distances may have to be used for comparing genomic sequences at different taxonomic levels.

Note that early analyses of genomic sequences with regard to similarities in the relative abundances of oligonucleotides of lengths *k*=1,…,6 exists and include [17–25]. Also, several alignment-free methods that use fixed-length word frequencies have been used for phylogenomic analysis of DNA sequences, [26–28]. These methods include statistical studies of word frequency within a DNA sequence [5, 29–34], or employ *k*-words and the Markov model to obtain information about DNA sequences [35–39]. Iterated map methods for DNA sequence comparison include CGR-based analyses, see [3, 40–46], and such alignment-free methods have been successfully applied for sequence comparison [4, 11, 12, 47–53].

The initial reports on CGRs of genomic sequences [3, 6] contained mostly qualitative assessments of CGR patterns of whole genes. In [54], several comparisons of eukaryotic genomic sequences, including within-species comparisons, were reported, using di-, tri-, and tetranucleotide relative abundance distance (*k*=2,3,4). In [25] di- and tetranucleotide abundance profiles (*k*=2,*k*=4) were compared for genomic collections from genomes of 5 gram-negative proteobacteria (including 2 complete genomes), 3 gram-positive bacteria, 2 mycoplasmas (complete genomes), 2 cyanobacteria (1 complete genome), and 3 thermophilic archaea (1 complete genome), using the *δ* ^∗^ distance which computes the average absolute difference of the dinucleotide relative abundance values. In [4], several datasets of up to 36 genomic DNA sequences were analyzed, and in [9] some various-length sequences were analyzed based on computing Euclidean distances between frequencies of their *k*-mers, for *k*=1,…,8. Subsequently, [10] computed the Euclidean distance between frequencies of *k*-mers (*k*≤5) for the analysis of 125 GenBank DNA sequences from 20 bird species and the American alligator. In [47], 27 microbial genomes were analyzed to find implications of 4-mer frequencies (*k*=4) on their evolutionary relationships. In [16], 20 mammalian complete mtDNA sequences were analyzed using the “similarity metric”, for *k*=7. In [50] a multigene dataset of 33 genes for 9 bacteria and one archaea species, as well as the whole genomes of a set of 16 *γ*-proteobacteria were analyzed, using values of *k* between 1 and 10, and Euclidean and *χ* ^2^ distances. In [11] a collection of 26 complete mitochondrial genomes was analyzed, using the Euclidean distance and an “image distance”, with a value of *k*=10. In [55] a megabase-scale phylogenomic analysis of the Reptilia was reported, that compared frequency distributions of 8-mer oligonucleotides (*k*=8) using Euclidean distance. Another study, [56], analyzed 459 bacteriophage genomes and compared them with their host genomes to infer host-phage relationships, by computing Euclidean distances between frequencies of *k*-mers for *k*=4. In [57], 75 complete HIV genome sequences were compared using the Euclidean distance between frequencies of 6-mers (*k*=6), in order to group them in subtypes. In [58] several datasets were analyzed (109 complete genomes of prokaryotes and eukaryotes, 34 prokaryote and chloroplast genomes, mitochondrial genomes of 64 vertebrates, and 62 complete genomes of alpha proteobacteria) using values of *k*=5,6 for protein-coding genes and *k*=11,12 for whole genomes, with two distances: chord distance and piecewise distance. In [12] a dataset of 3,176 complete mtDNA sequences was analyzed using an image distance, DSSIM, and a value of *k*=9, and several Molecular Distance Maps were obtained which displayed sequences’ interrelationships at several taxonomic levels (phylum Vertebrata, kingdom Protista, classes Amphibia-Insecta-Mammalia, class Amphibia, and order Primates).

The main contributions of this paper are:

- We tested and confirmed for an extensive dataset, of a total length of approximately 174Mbp, the hypothesis that CGR images of *genomic* DNA sequences can play the role of a *(graphic) genomic signature*, meaning that they have a desirable genome- and species-specificity. The dataset comprised 150 kbp fragments taken from genomes of six organisms, one from each of the six kingdoms of life. This was augmented by a set of 150 kbp fragments randomly sampled from all chromosomes of *M. musculus*, as a test-case of this method’s applicability at lower taxonomic levels.
- We assessed the performance of six different distances in this context, and this analysis included both same-genome and different-genome DNA fragment pairs. For several of these distances, the intragenomic values were overall smaller than intergenomic values, suggesting that this method could separate DNA genomic fragments belonging to different genomes, based on their CGRs.
- We showed that several distances outperform the Euclidean distance, which has so far been almost exclusively used for such studies. In particular, we determined that the DSSIM distance and the descriptor distance, adapted from computer vision for this application, were best able to differentiate sequences originating from different genomes at the kingdom level. Both these distances essentially compare the *k*-mer composition of DNA sequences (herein *k*=9).
- Based on preliminary data, we suggested the use of three-dimensional Molecular Distance Maps for improved visualization of the simultaneous interrelationships within a given set of genomic sequences.

Further analysis is needed to explore this method’s potential to differentiate genomic sequences originating from closely related species (e.g. within the same order). Additional refinements of the distances considered may have to be defined for optimal genomic DNA sequence identification and classification at very low taxonomic levels.

## Methods

In this section we first describe the dataset used for our analysis, then present an overview of the three main steps of the method, and conclude with a description of the six distances that we considered.

### Dataset

We used the complete genomes from six organisms, each representing one of the six kingdoms of life. For the first experiment, we used one complete chromosome from each genome, see Table 1. For additional information about the dataset see [59], Appendix B.

**Table 1:** Dataset for the first experiment: NCBI accession numbers of the complete chromosomes considered, in increasing order of their NCBI accession number

	Organism	NCBI Acc. Nr.
1	*H. sapiens*, chrom. 21 (Animalia)	NC_000021.8
2	*E. coli* (Bacteria)	NC_000913.3
3	*S. cerevisiae*, chrom. 4 (Fungi)	NC_001136.10
4	*A. thaliana*, chrom. 1 (Plantae)	NC_003070.9
5	*P. falciparum*, chrom. 14 (Protista)	NC_004317.2
6	*P. furiosus* (Archaea)	NC_018092.1

In order to have relatively comparable numbers of DNA sequences for each organism, we chose the longest chromosomes for all organisms except *H. sapiens*, for which the shortest chromosome was chosen.

The DNA sequences in the NCBI database are represented as strings of letters “A”, “C”, “G”, “T”, and “N” which represent the four nucleotides Adenine, Cytosine, Guanine, Thymine, and “unidentified Nucleotide”, respectively. For our analysis we ignored all letters “N”. In *S. cerevisiae* and *E. coli* there were no ignored letters, and in *P. falciparum* and *P. furiosus* the number of ignored letters is of the order of 0.001 *%* of the length of the sequence. In *H. sapiens* this number is 27 *%*, and in *A. thaliana* is 0.54 *%*. In *H. sapiens*, in particular, 96.4 *%* of these ignored letters exist in centromeric and telomeric regions of the chromosome.

The resulting genomic DNA sequences were divided into successive, non-overlapping, contiguous fragments, each 150 kbp long. When the last sequence was shorter than 150 kbp, it was not included in the analysis. This resulted in 234 fragments for *H. sapiens*, 30 fragments for *E. coli*, 10 fragments for *S. cerevisiae*, 201 fragments for *A. thaliana*, 21 fragments for *P. falciparum*, and 12 fragments for *P. furiosus*, for a total of 508 DNA fragments, see Table 2.

**Table 2:** The first experiment: Organisms considered, total length of the chromosome (respectively genome), number of ignored letters “N”, and number of DNA fragments (sequences) obtained by splitting a single complete chromosome per organism into consecutive, non-overlapping, equal length (150 kbp) contiguous fragments

Organism	Length(bp)	# Letters “N”	# Fragments
*H. sapiens*	48,129,895	13,023,253	234
*E. coli*	4,641,652	0	30
*S. cerevisiae*	1,531,933	0	10
*A. thaliana*	30,427,671	164,359	201
*P. falciparum*	3,291,871	37	21
*P. furiosus*	1,909,827	10	12

To maximize the diversity within each species, the dataset of the second experiment comprised fragments randomly sampled from each chromosome of the six chosen organisms, as follows. After deleting all “N” nucleotides, each chromosome was divided into successive, non-overlapping, contiguous fragments, each 150 kbp long. When the last fragment was shorter than 150 kbp, it was not included in the analysis. Next, for each chromosome we selected randomly 10 such fragments to represent the chromosome, see [59], Appendix B. In the cases where there were fewer than 10 fragments in a chromosome, all of them were considered. In the cases of *E. coli* and *P. furiosus*, we retained all complete fragments of the genome. This resulted in 240 fragments for *H. sapiens*, 30 fragments for *E. coli*, 73 fragments for *S. cerevisiae*, 50 fragments for *A. thaliana*, 121 fragments for *P. falciparum*, and 12 fragments for *P. furiosus*, for a total of 526 fragments.

### Overview

The method we used to analyze and classify genomic sequences has three steps: *(i)* generate graphical representations (images) of each DNA sequence using Chaos Game Representation (CGR), *(ii)* compute all pairwise distances between these images, and *(iii)* visualize the interrelationships implied by these distances as two- or three-dimensional maps, using Multi-Dimensional Scaling (MDS).

**Fig. 1:**
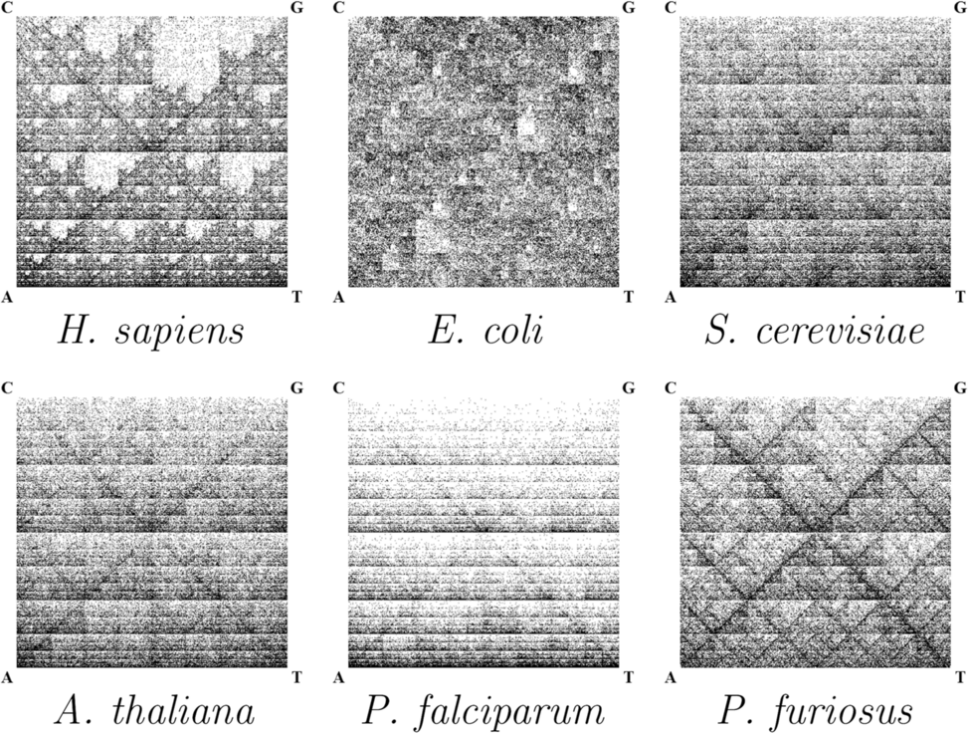
2^9^×2^9^ CGR images of 150 kbp genomic DNA sequences from *H. sapiens*, *E. coli*, *S. cerevisiae*, *A. thaliana*, *P. falciparum*, and *P. furiosus*

CGR is a method introduced by Jeffrey [3] in 1990 and studied in, e.g., [3, 6, 7, 11, 60–63] as a way to visualize the structure of a DNA sequence, This method associates an image to each DNA sequence as follows. Starting from a unit square with corners labelled *A, C, G,* and *T*, and the center of the square as the starting point, the image is obtained by successively plotting each nucleotide as the middle point between the current point and the corner labelled by the nucleotide to be plotted. If the generated square image has a size of 2^*k*^×2^*k*^ pixels, then every pixel represents a distinct *k*-mer: A pixel is black if the *k*-mer it represents occurs in the DNA sequence, otherwise it is white. CGR images of genetic DNA sequences originating from various species show patterns such as squares, parallel lines, rectangles, triangles, and also complex fractal patterns, Fig. 1.

For step *(i)*, a slight modification of the original CGR was used, introduced by Deschavanne [4]: a *k*-th order FCGR (frequency CGR) is a 2^*k*^×2^*k*^ matrix that can be constructed by dividing the CGR plot into a 2^*k*^×2^*k*^ grid, and defining the element *a*_*ij*_ as the number of points that are situated in the corresponding grid square. A second order FCGR is shown below, where *N*_*w*_ is the number of occurrences of the oligonucleotide *w* in the sequence *s*:

$$\begin{array}{@{}rcl@{}} FCGR_{2}(s) = \left(\begin{array}{cccc} N_{CC} & N_{GC} & N_{CG} & N_{GG} \\ N_{AC} & N_{TC} & N_{AG} & N_{TG} \\ N_{CA} & N_{GA} & N_{CT} & N_{GT} \\ N_{AA} & N_{TA} & N_{AT} & N_{TT} \\ \end{array} \right). \end{array} $$

The (*k*+1)-th order *F* *C* *G* *R*_*k*+1_(*s*) can be obtained by replacing each element *N*_*X*_ in *F* *C* *G* *R*_*k*_(*s*) with four elements

$$\begin{array}{@{}rcl@{}} \left(\begin{array}{cc} N_{CX} & N_{GX} \\ N_{AX} & N_{TX} \\ \end{array} \right) \end{array} $$

where *X* is a sequence of length *k* over the alphabet {*A, C, G, T*}.

For step *(ii)*, after computing the FCGR matrices for each of the 150 kbp sequences in a given dataset, the goal was to measure “distances” between every two CGR images. There are many distances that can be defined and used for this purpose, [64]. One of the goals of this study was to identify what distance is better able to differentiate the structural differences of various genomic DNA sequences and classify them based on the species they belong to. In this paper we use six different distances: Structural Dissimilarity Index (DSSIM), descriptor distance (adapted from computer vision for this application), Euclidean distance, Manhattan distance, Pearson correlation distance, and approximated information distance.

For step *(iii)*, after computing all possible pairwise distances we obtained six different distance matrices. To visualize the inter-relationships between sequences implied by each of the distance matrices, and to thus visually assess each of the distances, we used Multi-Dimensional Scaling (MDS). MDS is an information visualization technique introduced by Kruskal in [65]. MDS takes as input a distance matrix that contains the pairwise distances among a set of items (here the items are the 150 kpb DNA sequences analyzed). The output of MDS is a spatial representation of the items in a common Euclidean space, wherein each item is represented as a point and the spatial distance between any two points corresponds to the distance between the items in the distance matrix. Objects with a small pairwise distance will result in points that are close to each other, while objects with a large pairwise distance will become points that are far apart.

The combination of CGR/DSSIM/MDS was first proposed in [66], [12] as a tool to quantitatively measure and display the interrelationships among a set of complete mitochondrial sequences. The outputs of this method, called Molecular Distance Maps, are two-dimensional maps wherein each point represents a mitochondrial genome, and the spatial distances between any two points correspond to the differences between the structural composition of the corresponding DNA sequences. The ideal Molecular Distance Map is a placement of *n* items as points in an (*n*−1)-dimensional space. The two-dimensional Molecular Distance Map is simply an approximation, a flattening of this highly-dimensional space onto the plane, which may sometimes result in erroneous positioning of some points. Increasing the dimensionality of the Molecular Distance Map often results in a more accurate representation of the real interrelationships between sequences, as embodied in the original distance matrix.

### Distances

In this section we describe and formally define each of the six distances used in our analysis: DSSIM, descriptor distance (adapted from computer vision for this application), Euclidean, Manhattan, Pearson, and approximated information distance.

Structural Similarity Index, SSIM, was introduced in [13] for the purpose of assessing the degree of similarity between two images. Given two images *X, Y* as *n*×*n* matrices having as elements integers ranging in the interval [0,*L*], SSIM computes three factors (luminance, contrast and structure) and combines them to obtain a similarity value. However, instead of computing a global similarity between the two images, each image is divided into 11×11 sliding square windows *X* ^*ij*^(*Y* ^*ij*^respectively) with *i,j*=1,⋯,*n*−10 which move pixel by pixel to eventually cover the entire image. The SSIM similarity of any given pair of images is then computed by comparing their corresponding square windows. In addition, an 11×11 circular symmetric Gaussian weighting function $W \in \mathbb {R}^{11 \times 11}$ with a fixed standard deviation of 1.5, normalized to unit sum ($\sum _{p=1}^{11}\sum _{q=1}^{11} W_{\textit {pq}}=1$), is used. Then, the mean *μ*_*x,i,j*_ (*μ*_*y,i,j*_ for *Y*), variance *σ*_*x,i,j*_ (*σ*_*y,i,j*_ for *Y*) and correlation *σ*_*xy,i,j*_ are computed, as follows:

$$\begin{array}{@{}rcl@{}} \mu_{x,i,j}=\sum\limits_{p=1}^{11} \sum\limits_{q=1}^{11}W_{pq}X^{ij}_{pq} \end{array} $$

$$\begin{array}{@{}rcl@{}} \sigma_{x,i,j}=\sqrt{\sum\limits_{p=1}^{11} \sum\limits_{q=1}^{11}W_{pq}(X^{ij}_{pq}-\mu_{x,i,j})^{2}} \end{array} $$

$$\begin{array}{@{}rcl@{}} \sigma_{xy,i,j}=\sum\limits_{p=1}^{11} \sum\limits_{q=1}^{11} W_{pq}(X^{ij}_{pq}-\mu_{x,i,j})(Y^{ij}_{pq}-\mu_{y,i,j}) \end{array} $$

where *A*_*pq*_ denotes the (*p,q*) element of the matrix *A*. Based on these values, the luminance *l*(*X* ^*ij*^,*Y* ^*ij*^), contrast *c*(*X* ^*ij*^,*Y* ^*ij*^) and structure *s*(*X* ^*ij*^,*Y* ^*ij*^) are computed as

$$\begin{array}{@{}rcl@{}} l(X^{ij},Y^{ij})&=&\frac{2\mu_{x,i,j}\mu_{y,i,j}+C_{1}}{\mu_{x,i,j}^{2}+\mu_{y,i,j}^{2}+C_{1}}\\ c(X^{ij},Y^{ij})&=&\frac{2\sigma_{x,i,j}\sigma_{y,i,j}+C_{2}}{\sigma_{x,i,j}^{2}+\sigma_{y,i,j}^{2}+C_{2}}\\ s(X^{ij},Y^{ij})&=&\frac{\sigma_{xy,i,j}+C_{3}}{\sigma_{x,i,j}\sigma_{y,i,j}+C_{3}}\\ \end{array} $$

where *C*_1_=(0.01)^2^, *C*_2_=(0.03)^2^, $C_{3}=\frac {C_{2}}{2}$. Then, these three factors are combined to get

$$\begin{array}{@{}rcl@{}} SSIM(X^{ij},Y^{ij})=l(X^{ij},Y^{ij}) c(X^{ij},Y^{ij})s(X^{ij},Y^{ij}) \end{array} $$

and finally, the SSIM index used to evaluate the overall image similarity is computed as

$$\begin{array}{@{}rcl@{}} SSIM(X,Y)=\frac{1}{(n-10)^{2}}\sum\limits_{i=1}^{n-10}\sum\limits_{j=1}^{n-10} SSIM(X^{ij},Y^{ij}). \end{array} $$

In theory, the values for SSIM range in the interval [−1,1] with the similarity being 1 between two identical images, 0, for example, between a black image and a white image, and −1 if the two images are negatively correlated; that is, SSIM (*X,Y*)=−1 if and only if *X* and *Y* have the same luminance *μ* and every pixel *x*_*i*_ of image *X* has the inverted value of the corresponding pixel *y*_*i*_=2*μ*−*x*_*i*_ in *Y*.

To compute the distance rather than the similarity between two images, we calculate DSSIM (*X,Y*) = 1-SSIM (*X,Y*). Consequently, the range of DSSIM is the interval [0,2]: two identical images will result in a DSSIM distance of 0, while two images that are the negatives of each other would result in a DSSIM distance of 2.

For defining the *descriptor distance* we adapted for this application the spatial pyramid matching approach of [67], which is used to calculate hierarchical image descriptors. The *descriptor distance* between two FCGRs $X,Y \in \mathbb {N}^{2^{k} \times 2^{k}}$ aims to compare a combination of several different “descriptors”, that is, a combination of several different aspects, of the two given FCGRs.

A *descriptor* is a vector characterized by parameters *m* and *r*, as well as *r* intervals, where *m* is the size of the non-overlapping windows in which the FCGR is divided (scale of the comparison), and the *r* intervals represent the “granularity” of the analysis, in that they define the intervals of numbers of *k*-mer occurrences that are considered significant.

For a given *m*≤*k* and *r*, and intervals [*a*_0_,*a*_1_),[*a*_1_,*a*_2_),⋯,[*a*_*r*−1_,*a*_*r*_) such that $\bigcup _{i=0}^{r-1} [a_{i},a_{i+1})=[0,\infty)$ and [*a*_*i*_,*a*_*i*+1_)∩[*a*_*j*_,*a*_*j*+1_)=*∅*∀*i,j* with *i*≠*j*, a decriptor is constructed as follows.

Starting from the top-left corner, we divide each of the two FCGR matrices *X* and *Y* into non-overlapping submatrices of size 2^*m*^×2^*m*^. This procedure results in 4^*k*−*m*^ submatrices *X*_*ij*_ and *Y*_*ij*_ with *i,j*=1,⋯,2^*k*−*m*^, which will be pairwise compared.

The choice of the *r* intervals, called “bins”, points to the fact that, rather than considering the finest granularity, we are interested in a coarser comparison. This means that, instead of a computationally expensive pairwise comparison of all possible numbers of occurrences of *k*-mers, we are interested only in certain “bins” of such numbers. For example, in our case, we use *r*=5 and consider only 5 different bins, that is only *k*-mers with number of occurrences: 0 (not occurring), 1 (one occurrence), 2 (two occurrences), between 2 and 5, between 5 and 20, and greater than 20 (most frequent). Formally, we use *r*=5 and [0,*∞*)=[0,1)∪[1,2)∪[2,5)∪[5,20)∪[20,*∞*) as the 5 bins.

Afterwards, we compute for every *X*_*ij*_ a vector vec$X_{\textit {ij}} = \frac {1}{(2^{m} \times 2^{m})} (b_{1}, b_{2}, \cdots, b_{r})$ where *b*_*i*_=|{*x*∈*X*_*ij*_:*a*_*i*−1_≤*x*<*a*_*i*_}|. In our case, for each *X*_*ij*_, we compute a five-tuple wherein, for example, the 4th element represents the number of 9-mers whose number of occurrences is in the 4th bin, that is, at least 5 but less than 20. The division to 2^*m*^×2^*m*^ is to obtain a probability distribution for each submatrix. The same procedure is performed for *Y*_*ij*_, resulting in the vector vec *Y*_*ij*_.

We further append all vectors vec *X*_*ij*_ and form a new vector vec *X* ^*m, r*^ and, using the same order of appending, we append all vectors vec *Y*_*ij*_ forming a new vector vec *Y* ^*m, r*^. These two vectors are the “descriptors” of the FCGR matrices *X* and *Y* for the parameters *m*, *r* and the *r* chosen bins.

As a last step, we combine descriptors vec *X* ^*m, r*^ (respectively vec *Y* ^*m, r*^) for several values of *m* and *r* by appending them one after another, in the same order, to obtain the vector vec*X* (respectively vec*Y*).

The *descriptor distance* between the two FCGRs *X* and *Y* is now defined as the Euclidean distance between the vectors vec*X* and vec*Y*

$$\begin{array}{@{}rcl@{}} d_{D}(X,Y)= d_{E}(\text{vec}X, \text{vec}Y). \end{array} $$

In our case we computed descriptors for *m*=4,5,6 therefore forming vectors vec*X* and vec*Y* of length $5\left ((\frac {512}{64})^{2}+(\frac {512}{32})^{2}+(\frac {512}{16})^{2}\right)=6720$. In general, for a given *r*, the length of the vectors compared is $\phantom {\dot {i}\!} r ((2^{k-m_{1}})^{2} + (2^{k-m_{2}})^{2} + \ldots + (2^{k-m_{p}})^{2})$, where *m*_1_,*m*_2_,…,*m*_*p*_ are the values used for *m*. The choice of *m* for this study was made to balance the computational cost of calculating the vector of descriptors with the ability to compare the two matrices at various scales: large (*m*=6, that is, compare windows of size 64×64), medium (*m*=5, windows of size 32×32)) and small (*m*=4, windows of size 16×16). The parameter *r*=5 and the 5 bins were kept constant throughout our calculations but, in general, these parameters can also be varied, and the resulting vectors for each value added to the vector of descriptors, resulting in a larger vector.

In principle, the descriptor distance between two given FCGRs effectively compares the distribution of frequencies of *k*-mers between the corresponding submatrices *X*_*ij*_ and *Y*_*ij*_, and does that for several values of *m*, that is, at several different scales. (Note that, in each window *X*_*ij*_, all *k*-mers have the same suffix of length *k*−*m*.)

We now illustrate the *descriptor distance* by an example wherein *k*=3, *m*=2, *r*=3, and the 3 bins are [0,15)∪[15,30)∪[30,*∞*). Since *k*=3, the FCGR table will contain the number of occurrences of all 3-mers in a DNA sequence, as follows:



Take the two FCGRs $X, Y \in \mathbb {N}^{8 \times 8}$, (*k*=3, thus 2^3^×2^3^) corresponding to two genomic 150 kbp sequences of our dataset (one human and one bacterial), respectively. In order to use small numbers throughout the example, we divide all elements of the obtained matrices by 100 and take the integer part of each element, obtaining:

$$\begin{array}{@{}rcl@{}} X=\left(\begin{array}{cccccccc} 42 & 33 & 9 & 33 & 14 & 10 & 15 & 45 \\ 22 & 30 & 26 & 25 & 9 & 5 & 37 & 37 \\ 32 & 21 & 33 & 19 & 44 & 35 & 41 & 35 \\ 17 & 9 & 13 & 21 & 23 & 10 & 22 & 18 \\ 37 & 26 & 6 & 32 & 34 & 24 & 9 & 23 \\ 29 & 24 & 31 & 27 & 19 & 27 & 18 & 28 \\ 21 & 23 & 10 & 9 & 19 & 17 & 21 & 15 \\ 35 & 15 & 14 & 14 & 19 & 12 & 17 & 30 \\ \end{array} \right),\\ Y= \left(\begin{array}{cccccccc} 18 & 34 & 40 & 27 & 30 & 36 & 27 & 12 \\ 27 & 18 & 27 & 32 & 24 & 23 & 15 & 23 \\ 24 & 17 & 13 & 17 & 36 & 12 & 32 & 18 \\ 27 & 17 & 28 & 26 & 18 & 8 & 22 & 25 \\ 32 & 32 & 23 & 16 & 16 & 25 & 23 & 22 \\ 20 & 29 & 18 & 25 & 16 & 16 & 15 & 17 \\ 25 & 25 & 7 & 16 & 26 & 27 & 20 & 25 \\ 32 & 21 & 20 & 21 & 25 & 18 & 27 & 34 \\ \end{array} \right). \end{array} $$

Thus, in the human DNA sequence, the triplet CCC appears about 42 × 100 times, the triplet GCC appears about 33 × 100 times, the triplet CGC appears about 9 × 100 times, etc.

Since *m*=2, we divide each of the matrices *X* and *Y* into non-overlapping submatrices of size 4×4 (2^2^×2^2^). For *X* we thus obtain *X*_11_,*X*_12_,*X*_21_,*X*_22_

$$\begin{array}{@{}rcl@{}} \left(\begin{array}{cccc} 42 & 33 & 9 & 33 \\ 22 & 30 & 26 & 25 \\ 32 & 21 & 33 & 19 \\ 17 & 9 & 13 & 21 \\ \end{array} \right), \left(\begin{array}{cccc} 14 & 10 & 15 & 45 \\ 9 & 5 & 37 & 37 \\ 44 & 35 & 41 & 35 \\ 23 & 10 & 22 & 18 \\ \end{array} \right), \end{array} $$

$$\begin{array}{@{}rcl@{}} \left(\begin{array}{cccc} 37 & 26 & 6 & 32 \\ 29 & 24 & 31 & 27 \\ 21 & 23 & 10 & 9 \\ 35 & 15 & 14 & 14 \\ \end{array} \right), \left(\begin{array}{cccc} 34 & 24 & 9 & 23 \\ 19 & 27 & 18 & 28 \\ 19 & 17 & 21 & 15 \\ 19 & 12 & 17 & 30 \\ \end{array} \right). \end{array} $$

and similarly for *Y*.

Since the *r*=3 bins are [0,15)∪[15,30)∪[30,*∞*), we will count, for each submatrix, the number of 3-mers for which the number of occurrences is less than 15, between 15 and 30, and greater than or equal to 30. Thus we obtain vec$X_{11} = \frac {1}{16}(3, 7, 6)$ which has as elements the number of elements of *X*_11_ which belong in each of the intervals selected, divided by the total number of elements of *X*_11_. We proceed similarly for vec$X_{12} = \frac {1}{16} (5, 4,7)$, vec$X_{21} = \frac {1}{16} (5, 7, 4)$, vec$X_{22} = \frac {1}{16}(2, 12, 2)$ and we form vec*X* by appending these vectors one after the other, that is

$$\begin{array}{@{}rcl@{}} \text{vec}X = \frac{1}{16}\left(3, 7, 6, 5, 4, 7, 5, 7, 4, 2, 12, 2\right). \end{array} $$

We apply exactly the same procedure for the matrix *Y* and we get

$$\begin{array}{@{}rcl@{}} \text{vec}Y = \frac{1}{16} \left(1, 12, 3, 3, 9, 4, 1, 12,3,0, 15, 1\right). \end{array} $$

The descriptor distance between these two FCGRs is computed as the Euclidean distance between vec*X* and vec*Y*, in this case *d*_*D*_(*X,Y*)≈0.718. Note that, since we started by dividing the number of 3-mer occurrences by 100, as well as because of the bin selection, this is a fictitious example. The real value of the descriptor distance between the mentioned human and bacterial sequences is 8.66, and the range of the descriptor distance for this dataset of DNA sequences is [0, 13.17]. In general, the descriptor distance has a variable range, that depends on the choices of parameters used.

To compute the Euclidean, Manhattan and Pearson distances, we first convert the matrices $X, Y \in \mathbb {N}^{n \times n} $ into 1×*n* ^2^ vectors. For two vectors $x, y \in \mathbb {R}^{n}$, their Euclidean distance *d*_*E*_(*x, y*) and their Manhattan distance *d*_*M*_(*x, y*) are computed as

$$\begin{array}{@{}rcl@{}} d_{E}(x,y)=&\sqrt{\sum\limits_{i=1}^{n} (x_{i} -y_{i})^{2}},\\ d_{M}(x,y)=&\sum\limits_{i=1}^{n} |x_{i} -y_{i} |, \end{array} $$

while their Pearson distance *d*_*P*_(*x, y*) is defined as

$$\begin{array}{@{}rcl@{}} d_{P}(x,y)=1-\frac{\sigma_{xy}}{\sigma_{x} \sigma_{y}}, \end{array} $$

where

$$\begin{array}{@{}rcl@{}} \mu_{x}=\frac{1}{n}\sum\limits_{i=1}^{n} x_{i} \, \ \ \sigma_{x}= \sqrt{ \frac{1}{n-1} \sum\limits_{i=1}^{n}(x_{i}-\mu_{x})^{2} },\end{array} $$

$$\begin{array}{@{}rcl@{}} \sigma_{xy}=\frac{1}{n-1}\sum_{i=1}^{n} (x_{i}-\mu_{x})(y_{i}-\mu_{y}). \end{array} $$

In theory, the correlation coefficient $\frac {\sigma _{\textit {xy}}}{\sigma _{x} \sigma _{y}}$ ranges in the interval [−1,1], and therefore the Pearson distance ranges in the interval [0,2].

The last distance we considered is based on the information distance defined in [16]. The use of this distance is motivated computationally since it is easily computed from FCGRs as it tracks only the number of different *k*-mers for a sequence instead of the actual set. In [16], for a given *k*, the information distance for two strings *x,y* is defined as

$$\begin{array}{@{}rcl@{}} d_{AID}(x,y)=\frac{N_{k}(x|y)+N_{k}(y|x)}{N_{k}(xy)} \end{array} $$

with

$$\begin{array}{@{}rcl@{}} N_{k}(x|y)=N_{k}(xy)-N_{k}(x) \end{array} $$

where *N*_*k*_(*x*) is the number of different *k*-mers (possibly overlapping) which occur in *x*. We go one step further and modify this in order to avoid the creation of “unwanted” *k*-mers from the concatenation *xy* of *x* and *y*. We now show how to compute *N*_*k*_(*x*) for a sequence *x*. For a sequence *x*, first we build its FCGR$(x)=X \in \mathbb {N}^{2^{k} \times 2^{k}}$, which is a matrix of 2^*k*^×2^*k*^ with element values in $\mathbb {N}$. Then we unitize *X*, that is every non-zero entry becomes 1, while zeros remain 0. *N*_*k*_(*x*) is now computed as the sum of the elements of this unitized FCGR, that is, *N*_*k*_(*x*)=*f*(*X*)=SumOfElements(Unitize(*X*)). For two strings *x* and *y*, with FCGRs *X* and *Y* respectively, we define *N*_*k*_(*x*|*y*) as:

(1) $$\begin{array}{@{}rcl@{}}  N_{k}(x|y)= f(X+Y)-N_{k}(x) \end{array} $$

This slight modification of the information distance gives us also the desired properties of *d*(*x,x*)=0 and *d*(*x,y*)=*d*(*y,x*) which were not satisfied before. Using (1), we now define the *approximated information distance* (AID) as:

(2) $$\begin{array}{@{}rcl@{}}  d_{AID}(x,y)= 2-\frac{f(X)+f(Y)}{f(X+Y)} \end{array} $$

where *x,y* are the strings and $X,Y \in \mathbb {N}^{2^{k} \times 2^{k}}$ their FCGRs, respectively. It also turns out that this distance is in fact the normalized Hamming Distance of the unitized FCGRs *X* and *Y*. Note that, for two sets $\mathcal X$ and $\mathcal {Y}$, the normalized Hamming distance is $\frac {|\mathcal {X} \triangle \mathcal {Y}|}{|\mathcal {X} \cup \mathcal {Y}|} = 2 - \frac {|\mathcal {X}| + |\mathcal {Y}|}{|\mathcal {X} \cup \mathcal {Y}|}$ where △ denotes the symmetric difference.

Online Material, [59], includes the code used, the distance matrices, and an Appendix (Appendix A with details about accessing the online resources, Appendix B with information about the dataset, and Appendix C with additional histograms for the first experiment). The code, written in Wolfram Mathematica version 9, was used (and can be tested) for the generation of CGR images, the calculation of distance matrices, and the creation of 2D and 3D Molecular Distance Maps. The interactive webtool ModMap, [68], allows in-depth exploration of the 2D Mod Maps (Molecular Distance Maps) in this paper. When using the interactive webtool MoDMap, clicking on a distance underneath a dataset will result in plotting the MoD Map of the dataset computed with that distance. On any particular MoD Map, clicking on a point will display a window with information about the subsequence represented by that point: its NCBI accession number, scientific name of the organism it originates from, and its CGR pattern. Clicking on the “From here” and “To here” buttons on two such selected windows will display the distance between the corresponding genomic subsequences in the distance matrix.

## Results and discussion

For our dataset, we use *k*=9, that is, each DNA sequence was represented as a 2^9^×2^9^ FCGR matrix. In practice, this means that the FCGR of a DNA sequence contains the full information regarding its *k*-mer sequence composition, for *k*=1,2,…,9. The length choice of 150 kbp and value of *k*=9 is partly justified by the fact that, for a random sequence of length 150 kbp, its CGR at resolution 2^9^×2^9^ has around half of the pixels black, and half white, and partly justified by the fact that it empirically produced good results while at the same time being computationally inexpensive.

**Fig. 2:**
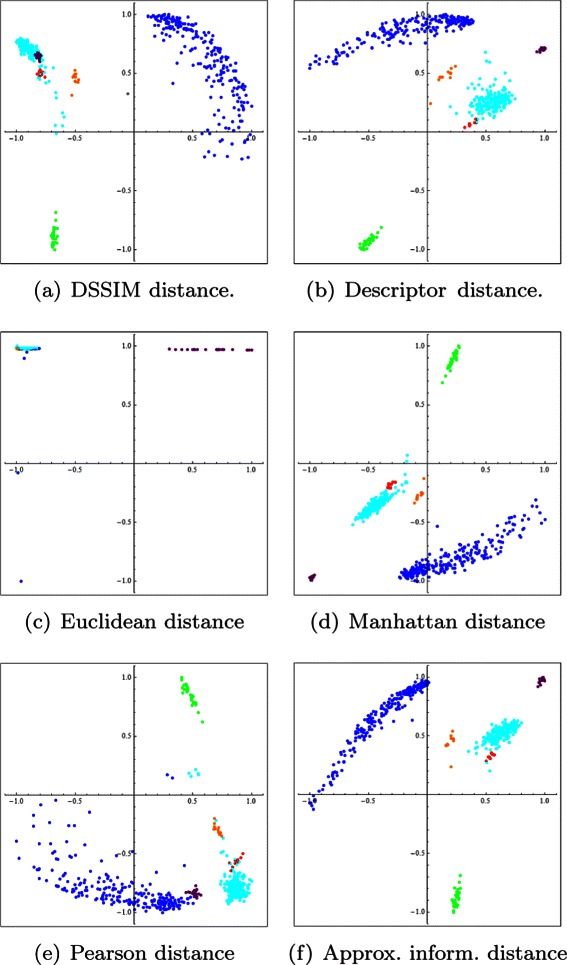
The first experiment: Two-dimensional Molecular Distance Maps of 150 kbp genomic sequences spanning one complete chromosome from each of six organisms, representing all kingdoms of life. The MoD Maps were obtained using (**a**) DSSIM, (**b**) descriptor, (**c**) Euclidean, (**d**) Manhattan, (**e**) Pearson and (**f**) approximated information distance, respectively. Each point corresponds to one 150 kbp genomic sequence from: *H. sapiens* (blue), *E. coli* (green), *S. cerevisiae* (red), *A. thaliana* (turquoise), *P. falciparum* (magenta), and *P. furiosus* (orange)

**Fig. 3:**
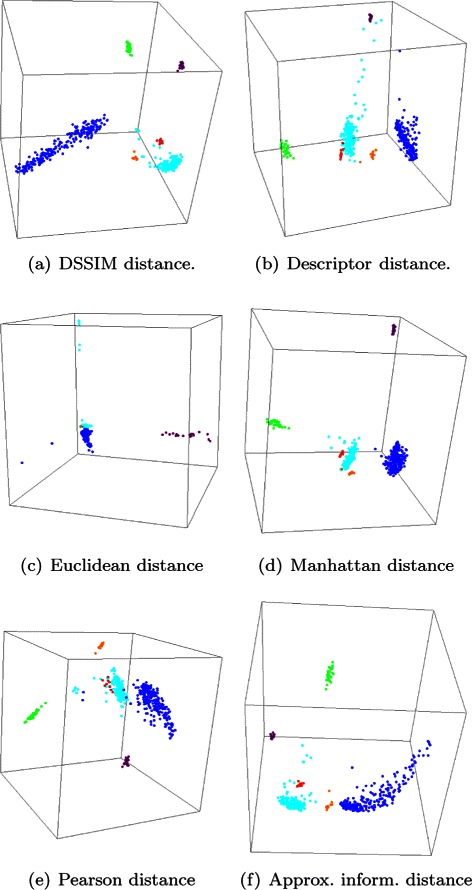
The first experiment: Three-dimensional Molecular Distance Maps of 150 kbp genomic sequences spanning one complete chromosome from each of six organisms, representing all kingdoms of life. The MoD Maps were obtained using (**a**) DSSIM, (**b**) descriptor, (**c**) Euclidean, (**d**) Manhattan, (**e**) Pearson and (**f**) approximated information distance, respectively. Each point corresponds to one 150 kbp genomic sequences from: *H. sapiens* (blue), *E. coli* (green), *S. cerevisiae* (red), *A. thaliana* (turquoise), *P. falciparum* (magenta), and *P. furiosus* (orange)

Figure 2 depicts two-dimensional Molecular Distance Maps obtained from the first experiment, using one complete chromosome for each organism, computed using the DSSIM distance, descriptor distance, Euclidean distance, Manhattan distance, Pearson distance and approximated information distance, respectively. Figure 3 depicts the corresponding three-dimensional Molecular Distance Maps for the same dataset. The projection of each three-dimensional map is chosen by hand in order to visually separate clusters of points which appear to be overlapping in the two-dimensional maps, as discussed below.

We note that MDS is not a clustering method, as the clusters are defined beforehand by the coloring scheme used (blue for *H. sapiens*, green for *E. coli*, and so on). MDS simply tries to display visually the interrelationships between the given items, based on the pairwise distances in the distance matrix which is its input. Note also that an increase in dimensionality from 2 to 3 can lead to a better cluster visualization. For example, if we compare the two-dimensional and the three-dimensional Molecular Distance Maps obtained using DSSIM, we see that points that appeared to be erroneously mixed with each other in the two-dimensional map, Fig. 2(a), (*S. cerevisiae* and *P. falciparum* sequences mixed in with *A. thaliana* sequences) are in fact clearly separated from each other in Fig. 3(a), the three-dimensional version of the Molecular Distance Map.

**Fig. 4:**
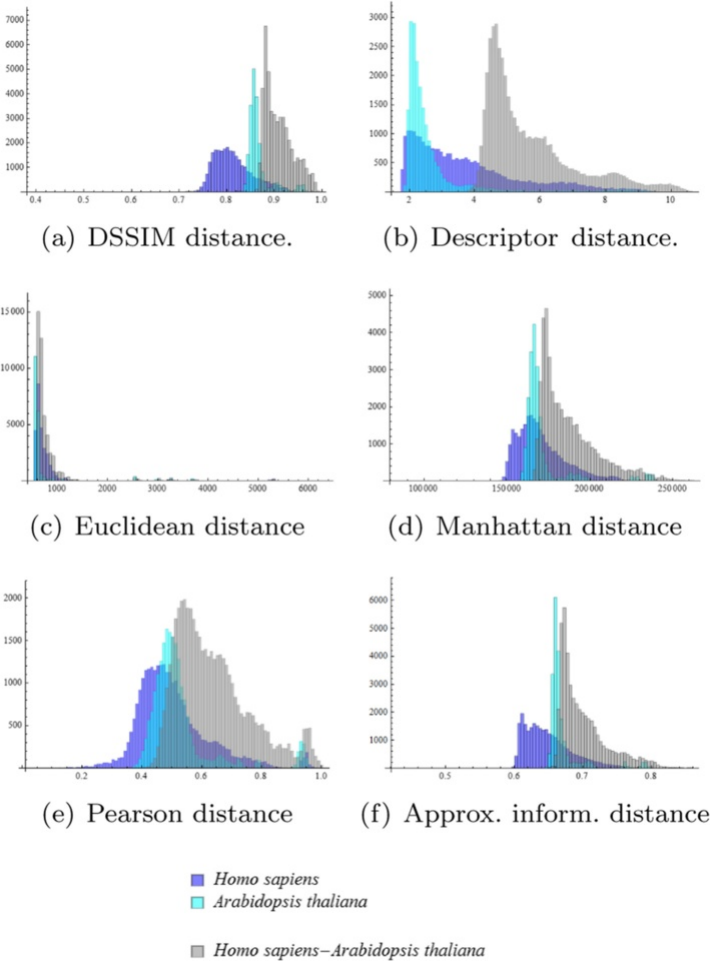
The first experiment (150 kbp fragments spanning one complete chromosome per each of the six organisms): Histograms of pairwise intragenomic and intergenomic distances (namely (**a**) DSSIM, (**b**) descriptor, (**c**) Euclidean, (**d**) Manhattan, (**e**) Pearson and (**f**) approximated information distance) among the DNA sequences from *H. sapiens* and *A. thaliana*. The histograms of intragenomic distances are coloured dark blue (*H. sapiens* - *H. sapiens*) and turquoise (*A. thaliana* - *A. thaliana*), while the histograms of intergenomic distances are coloured in grey (*H. sapiens* - *A. thaliana*)

Figure 4 displays the histograms of the pairwise intragenomic distances (dark blue and turquoise) and intergenomic distances (grey) of DNA sequences from *H. sapiens* and *A. thaliana*, obtained using each of the six distances. As noted, some distances seem to perform better than others. Visually, the poorest performer for these two sets of sequences (from *H. sapiens* and *A. thaliana*) seems to be the Euclidean distance wherein the intragenomic distances are as high as intergenomic distances, and no separation is visible. In contrast, DSSIM gives – for the same data – intergenomic distances that are overall much higher than intragenomic distances, resulting in a clear classification of DNA sequences into the species they belong to.

Table 3 displays the mean and standard deviation of distances between clusters *C*_*i*_ and *C*_*j*_, 1≤*i, j*≤6, where a cluster *C*_*ℓ*_ is defined as the set of all genomic sequences from the genome of organism *ℓ*, as labelled in Table 1. In each subtable, the diagonals represent the means and standard deviation for intragenomic distances, while the other entries are all intergenomic distances. From this table we see that for DSSIM, Manhattan and approximated information distance, the maximum of all the averages of intragenomic distances in this dataset is strictly smaller than the minimum of all the averages of intergenomic distances. For the descriptor distance and Pearson distance the previous statement does not hold but, for each pair of organisms, the two averages of intragenomic distances (e.g., *H. sapiens* - *H. sapiens* and *A. thaliana* - *A. thaliana*) are both lower than the average of the intergenomic distances (*H. sapiens* - *A. thaliana*). For the Euclidean distance, none of the previous statements holds: For example, the average of the *A. thaliana* - *A. thaliana* intragenomic distances (element 4-4 in the Euclidean distance subtable of Table 3) is 723, a value which is larger than 672, the average of the *S. cerevisiae* - *A. thaliana* intergenomic distances (element 3-4 in the Euclidean distance subtable of Table 3). The complete histograms of all pairwise comparisons *C*_*i*_−*C*_*j*_ can be found in [59], Appendix C.

**Table 3:** The first experiment: Mean and standard deviation of distances between clusters *C*_*i*_−*C*_*j*_ for *i,j*=1,…,6

-	1	2	3	4	5	6
1	0.81±0.04	0.99±0.01	0.92±0.02	0.91±0.03	0.92±0.03	0.91±0.02
2	-	0.85±0.01	0.97±0.01	0.99±0.01	0.99±0.01	0.99±0.
3	-	-	0.87±0.01	0.89±0.02	0.91±0.	0.91±0.01
4	-	-	-	0.87±0.03	0.9±0.02	0.91±0.01
5	-	-	-	-	0.74±0.01	0.94±0.
6	DSSIM					0.83±0.01
1	3.76±1.69	9.74±0.66	5.92±1.14	5.71±1.41	9.33±1.23	5.44±0.92
2	-	2.5±0.28	8.05±0.39	9.1±0.55	12.67±0.19	9.38±0.41
3	-	-	2.12±0.08	3.42±1.05	9.48±0.31	4.6±0.09
4	-	-	-	2.75±1.33	8.23±0.94	4.94±0.76
5	-	-	-	-	1.53±0.14	9.99±0.28
6	Descriptor					2.4±0.32
1	756±498	856±349	756±361	818±514	3914±510	812±356
2	-	558±5	674±17	802±366	4102±466	696±18
3	-	-	564±11	672±383	3964±472	633±20
4	-	-	-	723±535	3923±506	748±372
5	-	-	-	-	999±276	4085±468
6	Euclidean					585±24
1	171±15	222±5	189±13	188±17	213±20	191±9
2	-	175±2	209±4	219±8	252±4	218±3
3	-	-	171±2	177±10	206±2	184±2
4	-	-	-	172±16	200±11	188±9
5	-	-	-	-	105±3	224±2
6	Manhattan (in thousands)					167±3
1	0.5±0.12	0.97±0.02	0.69±0.1	0.64±0.12	0.65±0.09	0.81±0.06
2	-	0.71±0.02	0.93±0.02	0.96±0.02	0.98±0.01	0.99±0.02
3	-	-	0.6±0.02	0.6±0.07	0.71±0.03	0.75±0.02
4	-	-	-	0.53±0.11	0.63±0.09	0.76±0.04
5	-	-	-	-	0.02±0.01	0.94±0.01
6	Pearson					0.64±0.03
1	0.65±0.03	0.78±0.01	0.7±0.03	0.7±0.03	0.76±0.04	0.69±0.02
2	-	0.67±0.	0.75±0.01	0.77±0.02	0.85±0.01	0.77±0.01
3	-	-	0.67±0.01	0.68±0.02	0.74±0.	0.69±0.
4	-	-	-	0.67±0.03	0.73±0.02	0.69±0.02
5	-	-	-	-	0.64±0.01	0.76±0.01
6	Approx. Information					0.65±0.01

**Fig. 5:**
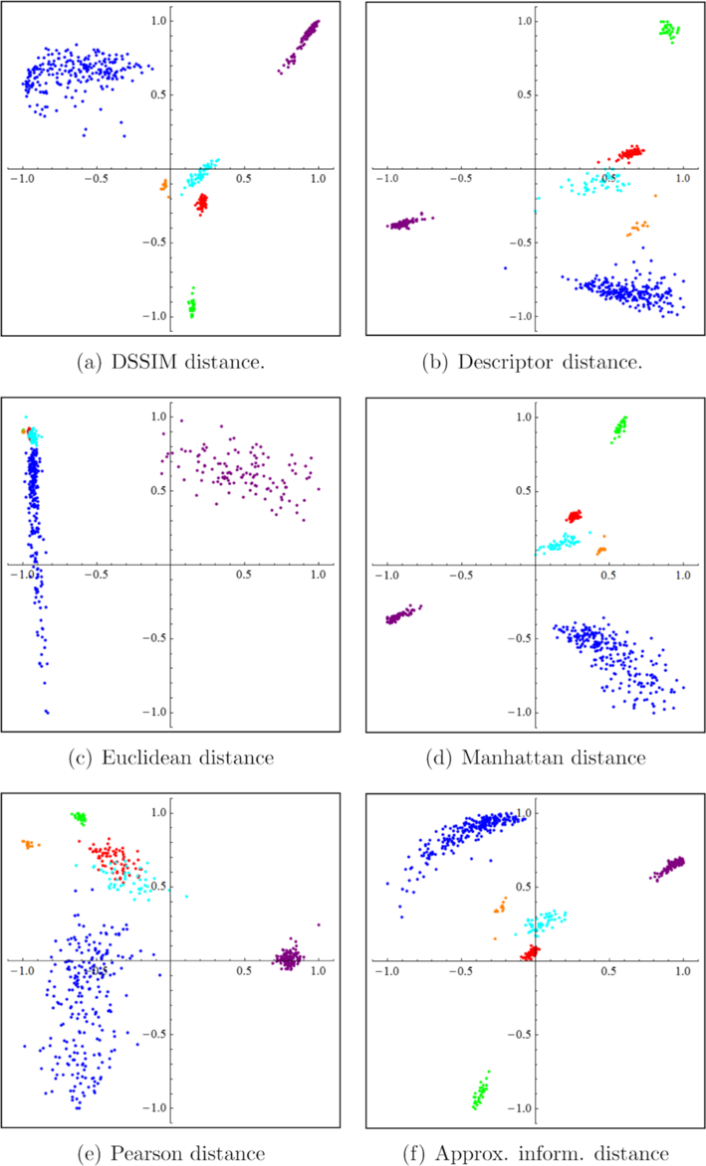
The second experiment: Two-dimensional Molecular Distance Maps of DNA genomic sequences sampled from the entire genomes of all six organisms, obtained using (**a**) DSSIM, (**b**) descriptor, (**c**) Euclidean, (**d**) Manhattan, (**e**) Pearson and (**f**) approximated information distance, respectively. The dataset consists of 10 randomly sampled fragments from each chromosome of multi-chromosome genomes, and all complete fragments from the genomes of *E. coli* and *P. furiosus*, for a total of 526 fragments. Each point corresponds to one such 150 kbp fragment from *H. sapiens* (blue), *E. coli* (green), *S. cerevisiae* (red), *A. thaliana* (turquoise), *P. falciparum* (magenta), and *P. furiosus* (orange)

**Fig. 6:**
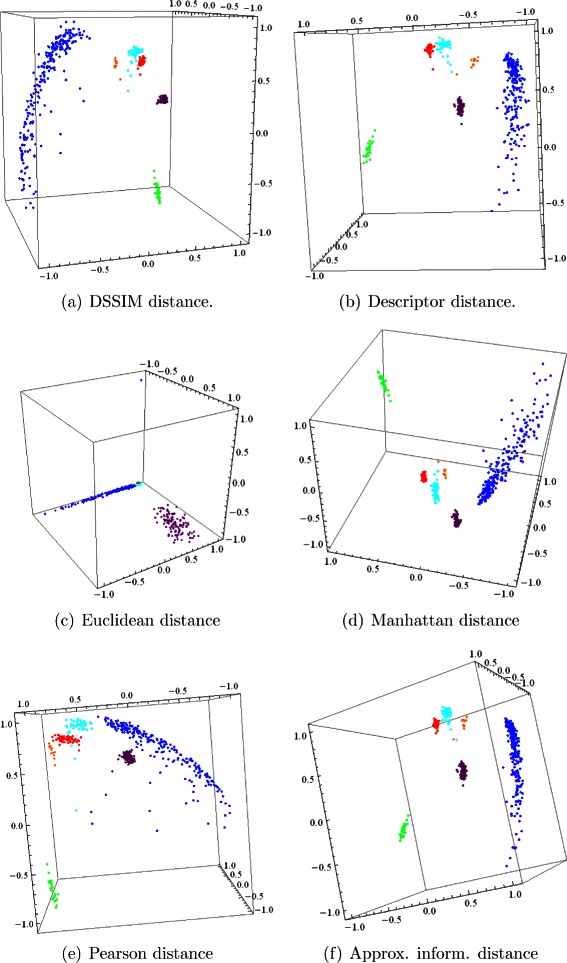
The second experiment: Three-dimensional Molecular Distance Maps of genomic DNA sequences sampled from the genomes of all six chosen organisms, obtained using (**a**) DSSIM, (**b**) descriptor, (**c**) Euclidean, (**d**) Manhattan, (**e**) Pearson and (**f**) approximated information distance, respectively. The dataset consists of 10 randomly sampled fragments from each chromosome of multi-chromosome genomes, and all complete fragments from the genomes of *E. coli* and *P. furiosus*, for a total of 526 fragments. Each point corresponds to one such 150 kbp fragment from *H. sapiens* (blue), *E. coli* (green), *S. cerevisiae* (red), *A. thaliana* (turquoise), *P. falciparum* (magenta), and *P. furiosus* (orange)

To maximize the diversity within each species, we performed a second experiment, with similar parameters as the first, but in which the fragments analyzed were randomly sampled from the entire genomes. The Molecular Distance Maps for this experiment are presented in Figs. 5 and 6. Note that the separation of sequences by the organism they belong to is even more clear than in the previous experiment, that used one complete chromosome from each organism. This suggests that (for this dataset), the CGR pattern is a genome-wide characteristic.

### Quality measures for distances

In this section we present three quality measures that each evaluates the quality of the six distances considered. In the data mining literature a wide range of quality measures for a given clustering has been defined; see for example [69, 70]. Most of these measures are designed to assess the quality of different automated clustering methods while using the same distance. Our set-up is different, as we use different distances while the clustering is fixed and given by the initial colour-coding of the sequence-representing points. Thus, we have to use other approaches to compare the distances we analyze. In particular, as the six distances have different ranges, we have to use assessment methods which are invariant to the scale of the distance.

The “ground-truth” that we use as a basis for our distance assessment is the fact that the “ideal” clustering of DNA sequences and the points that represent them is known: sequences from the same organism should be close to one another and far from sequences originating from other organisms. (This assumption is justified – for this dataset – as the six organisms considered are very different from one another, belonging to different kingdoms of life.) Thus, an optimal distance should yield a relatively small value for two FCGRs which were generated from the DNA sequences originating from the same organism, and relatively high values for two FCGRs originating from DNA sequences coming from different organisms.

In order to assess each of the six distances quantitatively, we computed three quality measures which rate different features of a distance:

- the correlation to an idealized cluster distance
- the silhouette cluster accuracy
- the relative overlap between the intragenomic and intergenomic distance histograms.

Let us stress that all three quality measures of the six distances are based on the distance matrices which we computed and not on their MDS plots. We will define the three quality measures such that their expected values range in the interval [0,1] where higher values correspond to better performance.

Let us first describe the three quality measures informally. An idealized distance is a distance that would be able to differentiate DNA sequences by species, that is, a distance *δ* for which *δ*(*x,y*)=0 if *x* and *y* are sequences from the same species and *δ*(*x,y*)=1 otherwise. The first quality measure, the *correlation to an idealized cluster distance*, measures how well a distance is linearly correlated to the idealized distance *δ*. The second quality measure, *silhouette cluster accuracy*, is the percentage of points that are best embedded in the cluster they belong to. The third quality measure quantifies the “visual overlap” between the intragenomic and intergenomic distance histograms. Given our dataset, it is reasonable to expect that a good distance gives a low value if applied to FCGRs of genomic sequences of the same organism, and a high value when applied to FCGRs of genomic sequences from two different organisms, thus separating the histograms of intragenomic distances from that of intergenomic distances. This is illustrated by the histograms in Fig. 4, where a high overlap between the graph of intragenomic distances (dark blue and turquoise) and the graphs of intergenomic distances (grey) is an indication of a poorly performing distance. In a theoretically optimal situation, there would exist a value *c* such that all distances that are smaller than *c* are intragenomic distances and all distances that are larger than *c* are intergenomic distances. This can usually not be expected from real data, but a low overlap between histograms is nevertheless indicative of a “good” distance.

In order to formally define the three quality measures, we consider a dataset *V* which is partitioned into *p* non-overlapping clusters *C*_1_,…,*C*_*p*_ for which a distance $d_{\alpha }\colon V\times V \to \mathbb {R}_{\ge 0}$ exists. The cardinalities of the sets are |*V*|=*m* and |*C*_*i*_|=*m*_*i*_ for *i*=1,…,*p*. In our analysis, *p*=6 and *C*_1_ contains all FCGRs generated from genomic DNA sequences from *H. sapiens*, *C*_2_ contains all FCGRs generated from genomic sequences of *E.coli*, and so on, according to the order in Table 1. The distance *d*_*α*_ is one of the six distances *α*∈{DSSIM, D, E, M, P, AID }.

The *correlation to an idealized cluster distance* is computed as follows. We define the *idealized cluster distance* as a function (or matrix) *δ*:*V*×*V*→{0,1} such that *δ*(*x,y*)=0 if and only if *x* and *y* belong to the same cluster, and *δ*(*x,y*)=1 otherwise. Because we can view *d*_*α*_ and *δ* as discrete, symmetric functions which have the same domain, we can compute their correlation coefficient. We define the correlation of *δ* to *d*_*α*_ to be the Pearson correlation of *δ* and *d*_*α*_. More precisely, the upper triangular part of the matrix corresponding to a distance *d*_*α*_ is interpreted as a vector (*x*_1_,…,*x*_*n*_) and compared with the corresponding values (*y*_1_,…,*y*_*n*_) given by *δ*. We obtain the *δ*-correlation as

$$\begin{array}{@{}rcl@{}} \mathcal{D}_{\alpha} = \frac{\sigma_{xy}}{\sigma_{x}\sigma_{y}}. \end{array} $$

The correlation ranges in the interval [−1,1]: a value of 1 means that *d*_*α*_ and *δ* are linearly correlated, and a value of 0 means that they are unrelated. In other words, if the value obtained by measuring the *correlation* of a given distance *to the idealized cluster distance* is close to 1, this means that the given distance is closer to the idealized cluster distance, and hence, performs well. Note that negative values for this measure are not expected as this would imply that *d*_*α*_ and *δ* were negatively related (*d*_*α*_ would perform worse than a matrix containing random entries).

The *silhouette cluster accuracy* is based on the *silhouette coefficient* defined in [71] as a measure that determines how well a single point is embedded in the cluster to which it belongs. For a point *x* from cluster *C*_*i*_ we define *a*_*x*_ as the average distance of this point to all other points in *C*_*i*_, that is,

$$\begin{array}{@{}rcl@{}} a_{x} = \frac1{m_{i}-1}\sum\limits_{y\in C_{i},y\neq x} d_{\alpha}(x,y), \end{array} $$

and we define *b*_*x*_ as the minimum over the average distances of *x* to all points of a different cluster

$$\begin{array}{@{}rcl@{}} b_{x} = \min_{j=1,j\neq i}^{K} \left\{ \frac1{m_{j}}\sum\limits_{y\in C_{j}} d_{\alpha}(x,y) \right\}. \end{array} $$

The silhouette coefficient of *x* is defined as

$$\begin{array}{@{}rcl@{}} \mathcal{S}_{\alpha}(x) = \frac{b_{x} - a_{x}}{\max\{a_{x},b_{x}\}}. \end{array} $$

If a point *x* has a silhouette coefficient $\mathcal {S}_{\alpha }(x) \le 0$, then *x* is at least as close to a cluster to which it does not belong than to its own cluster. The *silhouette cluster accuracy* $\mathcal {A}_{\alpha }$ denotes the percentage of points with a silhouette coefficient greater than 0, that is the percentage of points which are well-embedded in their own cluster,

$$\begin{array}{@{}rcl@{}} \mathcal{A}_{\alpha} = \frac{\left| \{ x\in V \mid \mathcal{S}_{\alpha}(x) > 0 \} \right|}{m}. \end{array} $$

Obviously, the silhouette cluster accuracy ranges in [0,1] with a high accuracy being desirable.

For assessing the *relative overlap* of the histograms, consider any two clusters *C*_*i*_ and *C*_*j*_ with *i*≠*j* (for example, *C*_1_ is the *H. sapiens* cluster and *C*_4_ the *A. thaliana* cluster). We compare the two sets of intragenomic distances *C*_*i*_– *C*_*i*_ and *C*_*j*_– *C*_*j*_ with the set of intergenomic distances *C*_*i*_– *C*_*j*_. For a distance *d*_*α*_, we divide the range from min(*d*_*α*_) to max(*d*_*α*_) in this dataset into 100 bins of size $r = \frac {\max (d_{\alpha }) - \min (d_{\alpha })}{100}$ and count the distances which fall into this bin: *c*_*i,i*_[*ℓ*] denotes bin *ℓ* containing distances from *C*_*i*_– *C*_*i*_ and *c*_*i,j*_[*ℓ*] denotes bin *i* containing distances from *C*_*i*_– *C*_*j*_. For *ℓ*=1,…,100 we let

$$\begin{array}{@{}rcl@{}} c_{i',j'}[\ell] = &| \{ \{x,y\}\mid x\in C_{i'},y\in C_{j'} \text{ and } x \neq y \\ &\text{and } (\ell-1)\cdot r < d_{\alpha}(x,y) \le \ell\cdot r \} |. \end{array} $$

By $\phantom {\dot {i}\!}s_{i',j'}$ we denote the sum over all $\phantom {\dot {i}\!}c_{i',j'}$-bins, that is, $\phantom {\dot {i}\!}s_{i',j'} = \sum _{\ell =1}^{100} c_{i',j'}[\ell ]$. We define the relative overlap $\mathcal {O}_{\alpha }(i,j)$ of *C*_*i*_– *C*_*i*_ (intragenomic distances) with *C*_*i*_– *C*_*j*_ (intergenomic distances) as

$$\begin{array}{@{}rcl@{}} \mathcal{O}_{\alpha}(i,j) = \frac{\max\{s_{i,i}, s_{i,j} \}} {\min\{s_{i,i}, s_{i,j} \}} \cdot \frac{\sum_{i=1}^{100} \min\{c_{i,i},c_{i,j}\}} {\sum_{i=1}^{100} \max\{c_{i,i},c_{i,j}\}}. \end{array} $$

The relative overlap $\mathcal {O}_{\alpha }(j,i)$ of *C*_*j*_– *C*_*j*_ with *C*_*i*_– *C*_*j*_ is defined analogously; note that $\mathcal {O}_{\alpha }(i,j) \neq \mathcal {O}_{\alpha }(j,i)$ in general. The overlap is normalized to the range [0,1] where 0 means no overlap of elements of bins between intra- and intergenomic distances, and 1 means that one of the histograms completely “covers” the other. Also note that we are not interested in the overlap of *C*_*i*_– *C*_*i*_ with *C*_*j*_– *C*_*j*_ as both sets of distances are intragenomic distances.

Since we intend to define a quality measure where a value close to 1 should represent a small overlap, we will use $1-\mathcal {O}_{\alpha }(i,j)$. Furthermore, we combine these quantities for all possible pairs of clusters *C*_*i*_ and *C*_*j*_, obtaining *the relative overlap* as:

$$\begin{array}{@{}rcl@{}} \mathcal O_{\alpha} = 1 - \frac{1}{p(p-1)} \sum\limits_{i=1}^{p}\sum\limits_{j=1,i\neq j}^{p} \mathcal O_{\alpha}(i,j). \end{array} $$

For example, in Fig. 4, for each of the considered distance, the dark blue histograms depict the *C*_1_−*C*_1_ (*H. sapiens* – *H. sapiens*) intragenomic distances, the turquoise histograms the *C*_4_−*C*_4_ (*A. thaliana* – *A. thaliana*) intragenomic distances, and grey histograms the *C*_1_−*C*_4_ (*H. sapiens* – *A. thaliana*) intergenomic distances. As seen from this figure, the descriptor distance appears to visually perform best at separating the two intragenomic distance histograms from the intergenomic histogram, while the Euclidean distance has the weakest performance. The relative overlap attempts to quantify this by computing the overlaps of each of the two pairs of histograms (dark blue with grey, and turquoise with grey). Note that small visual histogram overlaps will result in a high numerical *relative overlap*, and is indicative of a better performing distance.

### Distance comparison results

For the first experiment (one complete chromosome from each organism) the results of ranking the six distances, using the three quality measures, are listed in Table 4. Recall that all quality measures have an expected range of [0,1] where larger values imply better performance.

**Table 4:** The first experiment: Summary of quality measures for the performances of six distances (DSSIM, descriptor, Euclidean, Manhattan, Pearson, approximated information distance) on a dataset of 508 genomic DNA sequences spanning one complete chromosome for multi-chromosomes organisms and the complete genome otherwise, of one organism from each kingdom of life

	$\mathcal {D}_{\alpha }$	$\mathcal {A}_{\alpha }$	$\mathcal {O}_{\alpha }$	*z*-score sum	Rank
DSSIM	0.627	1.000	0.965	1.895	2nd
Descriptor	0.639	0.976	0.988	2.509	1st
Euclidean	0.231	0.325	0.907	−4.831	6th
Manhattan	0.527	1.000	0.951	0.84	3rd
Pearson	0.536	0.980	0.888	−0.875	5th
Approx. Inf.	0.527	1.000	0.937	0.462	4th

$\mathcal {D}_{\alpha }$ is the correlation to an idealized cluster, $\mathcal {A}_{\alpha }$ the silhouette cluster accuracy, and $\mathcal {O}_{\alpha }$ the relative overlap. Higher is better

To compare each distance relative to all the other distances, we compute for each quality measure (each column) the *standard scores* (*z*-scores) of each distance *d*_*α*_, where *α*∈{DSSIM, D, E, M, P, AID }, as $z(d_{\alpha }) = \frac {d_{\alpha } - \mu }{\sigma }$ where *μ* is the mean and *σ* is the deviation for that particular quality measure (column).

A positive value of the standard score will mean that a distance performs above average (in this category) and a negative value that it performs below average. Finally, we compute the sum of the *z*-scores for each quality measure as seen in Table 4, second last column. Note that the total of *z*-scores for a distance represents the performance of that distance relative to the other distances, and indicates its relative ranking.

Table 5 contains the results of the distance comparison for the second experiment, that sampled 10 fragments from each chromosome. Interestingly, the ranking of distances is the same for both experiments.

**Table 5:** The second experiment: Summary of quality measures for the performances of six distances (DSSIM, descriptor, Euclidean, Manhattan, Pearson, approximated information distance) on a dataset of 526 genomic DNA sequences sampled randomly (10 fragments per chromosome for multi-chromosome organisms, and all fragments of the genome otherwise) from the genomes of organisms from each kingdom of life

	$\mathcal {D}_{\alpha }$	$\mathcal {A}_{\alpha }$	$\mathcal {O}_{\alpha }$	*z*-score sum	Rank
DSSIM	0.729	1.000	0.964	1.980	2nd
Descriptor	0.726	0.998	0.984	2.336	1st
Euclidean	0.438	0.608	0.861	−5.292	6th
Manhattan	0.662	1.000	0.955	1.172	3rd
Pearson	0.639	0.949	0.875	−0.954	5th
Approx. Inf.	0.637	1.000	0.946	0.759	4th

$\mathcal {D}_{\alpha }$ is the correlation to an idealized cluster, $\mathcal {A}_{\alpha }$ the silhouette cluster accuracy, and $\mathcal {O}_{\alpha }$ the relative overlap. Higher is better

The conclusion of these analyses is that the best performing distances for this dataset are the descriptor distance and DSSIM. The Manhattan, Pearson, and approximate information distances perform well in some categories but not so well in other categories. For this dataset and value of *k*, the Euclidean distance had the weakest performance in all measured categories, which confirms the visual assessment of the MDS plots obtained by using the Euclidean distance, as seen in Figs. 2 and 3.

It is worth noting that the two distances which perform best (DSSIM and descriptor) treat FCGR matrices as two-dimensional maps in which the local arrangement of the cells (matrix entries) influences the computed distance, whereas the other distances treat the FCGR matrices as linear vectors. This suggests that the organization of the *k*-mer tallies (in this paper *k*=9) of a DNA sequence as an FCGR matrix, rather than a simple vector, reveals structural properties of the DNA sequence that could be utilized in order to identify and classify genomic DNA sequences.

## Conclusions

In this study we test, at the kingdom level, the hypothesis that CGR-based genomic signatures of genomic DNA sequences are indeed species and genome-specific. With this goal in mind we first analyzed over five hundred 150 kbp DNA genomic sequences spanning one complete chromosome from each of six organisms, representing all kingdoms of life. We then separately analyzed over five hundred 150 kbp genomic sequences randomly sampled from the complete genomes of all organisms considered.

Our quantitative comparison of six different distances suggests that several other distances outperform the Euclidean distance, which has been until now almost exclusively used in such studies. Our preliminary results show that two of these distances, DSSIM and descriptor distance (introduced here) when applied to CGR-based genomic signatures, have indeed the ability to differentiate between DNA sequences coming from different species at this taxonomic level. This indicates that the *k*-mer sequence composition (where *k*=1,2,…,9) of genomic sequences contains taxonomic information which could potentially aid in the identification, comparison and classification of species based on molecular evidence. The two-dimensional and three-dimensional Molecular Distance Maps we obtain, which visualize the simultaneous intragenomic and intergenomic interrelationships among the sequences in our dataset, show this method’s potential.

Further analysis is needed to explore this method’s applicability to the genomic species identification and classification at lower taxonomic levels. As a preview experiment, we applied it to 240 fragments, randomly sampled from the entire genome of *H. sapiens* (10 fragments per chromosome), and 210 fragments randomly sampled from the entire genome of *M. musculus* (10 fragments per chromosome). See [59], Appendix B, for dataset details.

**Fig. 7:**
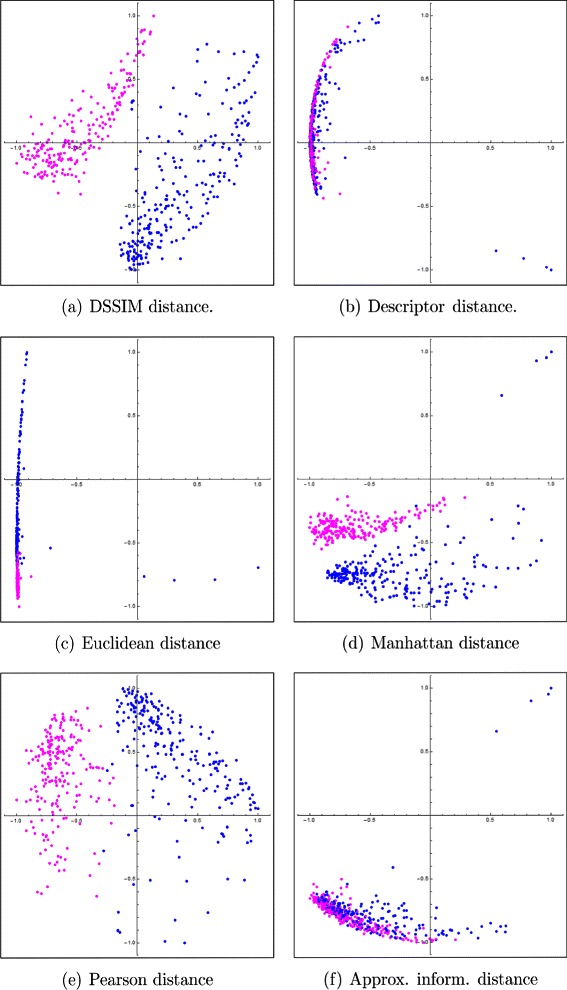
The preview experiment: Two-dimensional Molecular Distance Maps of 150 kbp genomic DNA sequences, randomly sampled from each chromosome (10 fragments per chromosome) of *H. sapiens* (blue), *M. musculus* (fuchsia) using (**a**) DSSIM, (**b**) descriptor, (**c**) Euclidean, (**d**) Manhattan, (**e**) Pearson and (**f**) approximated information distance, respectively

**Fig. 8:**
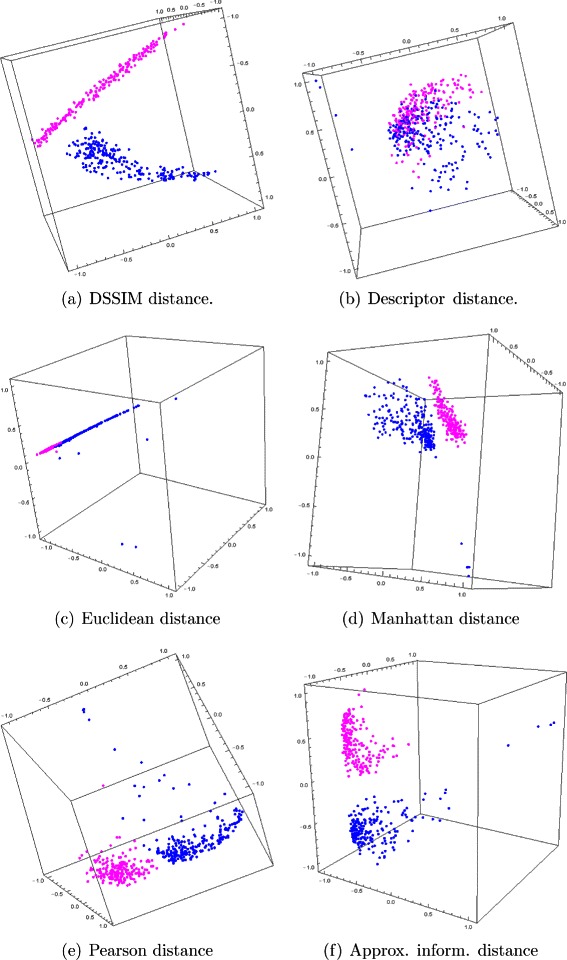
The preview experiment: Three-dimensional Molecular Distance Maps of 150 kbp genomic DNA sequences, randomly sampled from each chromosome (10 fragments per chromosome) of *H. sapiens* (blue), *M. musculus* (fuchsia) using (**a**) DSSIM, (**b**) descriptor, (**c**) Euclidean, (**d**) Manhattan, (e) Pearson and (**f**) approximated information distance, respectively

The Molecular Distance Maps of these 450 DNA sequences, 150 kbp each (see Figs. 7 and 8) suggest that several of the distances are able to differentiate between DNA sequences at lower taxonomic levels. As seen in Table 6, the Euclidean distance was again outperformed by other distances, when assessed with the quality measures we described. However, we note a change in the distance rankings, with Pearson and DSSIM ranking first and respectively second, and the descriptor distance ranking last. This may be because the descriptor distance is able to identify large pattern-differences between CGR images, which may be more suitable when comparing genomic sequences at high taxonomic levels, while DSSIM is good at picking up subtle differences between similar CGR images and thus it may be better suited to comparing genomic sequences from more closely related species. Overall, this suggests that different distances may have to be chosen, depending on the taxonomic level of the analysis.

**Table 6:** The preview experiment: Summary of quality measures for the performances of six distances (DSSIM, descriptor, Euclidean, Manhattan, Pearson, approximated information distance) on a dataset of 450 DNA sequences, sampled from the entire genome (10 fragments per chromosome) of *H. sapiens* and *M. musculus*

	$\mathcal {D}_{\alpha }$	$\mathcal {A}_{\alpha }$	$\mathcal {O}_{\alpha }$	*z*-score sum	Rank
DSSIM	0.422	1.000	0.618	3.014	2nd
Descriptor	0.032	0.560	0.063	−3.347	6th
Euclidean	0.079	0.658	0.318	−1.558	4th
Manhattan	0.209	0.969	0.336	0.601	3rd
Pearson	0.531	0.993	0.647	3.643	1st
Approx. Inf.	0.101	0.578	0.195	−2.353	5th

$\mathcal {D}_{\alpha }$ is the correlation to an idealized cluster, $\mathcal {A}_{\alpha }$ is the silhouette cluster accuracy, and $\mathcal {O}_{\alpha }$ is the relative overlap. Higher is better

Further large-scale computational experiments have to be carried out to confirm these preliminary results and establish their validity, as well as to establish the applicability of this method to genomic sequences identification and classification at lower taxonomic levels. Such experiments could provide additional insights regarding the choice of optimal distance for structural genomic sequence comparisons in different settings.
